# Single-nucleus transcriptomics illuminates sex differences during murine Escherichia coli pyelonephritis

**DOI:** 10.21203/rs.3.rs-6976359/v1

**Published:** 2025-10-31

**Authors:** David Hunstad, Teri Hreha, Abigail Manson, Christina Collins, Haojia Wu, Christophe Georgescu, Benjamin Humphreys, Ashlee Earl

**Affiliations:** Washington University in St. Louis; Washington University at St. Louis; Broad Institute; Washington University School of Medicine; Washington University in St. Louis; Broad Institute; Washington University School of Medicine; Broad Institute

## Abstract

There are profound sex differences in the prevalence and outcomes of urinary tract infections (UTI). While females comprise the majority of infections, males exhibit higher morbidity and mortality with upper-tract UTI. Correspondingly, preclinical modeling has demonstrated that male and androgen-exposed female mice are highly susceptible to severe high-titer pyelonephritis, a phenotype observed in < 20% of females. We subjected kidneys from female, male, and androgen-exposed female C3H/HeN mice exhibiting high-titer pyelonephritis and PBS-exposed control mice to single-nucleus RNA sequencing, creating, to our knowledge, the first whole-kidney single-nucleus transcriptomic dataset reflecting an infected state. We differentiated healthy cell populations from those affected during UTI and showed sex-discrepant responses that extended to kidney cell types beyond those directly interacting with bacteria. The female response to UTI comprised a more limited range of cell types exhibiting significant upregulation of genes within KEGG pathways and transcription factor regulons related to pro-inflammatory processes. Meanwhile, males evidenced predisposition to injury pathways even with control (saline) inoculation and responded to UTI with less intensity but across more cell types than females. In total, these data illuminate sex-discrepant transcriptional responses and outcomes in renal infection and enable extended dissection of these responses at the cellular and molecular level.

## INTRODUCTION

Urinary tract infections (UTIs) affect millions of people worldwide, resulting in millions of dollars spent in hospital visits and treatments^1^. Over 75% percent of UTIs are caused by uropathogenic *Escherichia coli* (UPEC)^1^, infecting the bladder (cystitis) and often ascending to the kidneys (pyelonephritis). Approximately 50% of women will experience a UTI in their lifetime, and approximately 25% of those women experience recurrent infections^2^. Although UTI in males is less common, elderly men experience UTI at rates similar to those in women, and among infants less than 6 months of age, UTI incidence in boys outpaces that in girls^3^. Further, upper-tract UTIs in men carry increased morbidity and mortality^3,4^.

When UPEC is inoculated into the bladders of female C3H/HeN mice (a background exhibiting vesicoureteral reflux [VUR], a major risk factor for pyelonephritis in children), 70–80% of these females resolve infection within 7 days after inoculation, while 20–30% experience chronic high-titer infection in both the bladder and kidneys^5^. In mice that resolve an initial UTI, bladder epithelial remodeling increases the rate of chronicity upon reinfection to ~ 60%^5^, while little is known about the cellular and molecular mechanisms underlying chronic infection of the female kidney. In contrast, virtually all male and most androgen-exposed female C3H/HeN mice develop persistent high-titer cystitis and pyelonephritis after primary inoculation, in an androgen receptor-dependent manner^6–8^. Most of these mice exhibit renal abscesses, characterized by abundant neutrophil infiltration and leaving renal scars^6–9^. Such abscesses are regional, and the kidneys display zones of both healthy and injured tissue^7^. This distribution complicates studies of the renal response to UTI, especially at early time points, as whole-kidney analyses tend to be insensitive to changes that are occurring only near foci of infection.

Extensive work has been published using single-nucleus RNA sequencing (snRNAseq) in models of non-infectious acute kidney injury (AKI)^10–13^, enabling a deeper understanding of the renal response to injury, including mechanisms of cellular regeneration and failed repair. Here, in the context of this single-cell work in AKI, we profiled sex-specific host responses to renal infection. We studied kidneys from UPEC-infected female, male, and androgen-exposed female C3H/HeN mice at 5 days post infection (dpi), along with PBS-inoculated control mice, to better understand the effect of host sex on development of pyelonephritis. This time point was chosen because renal outcomes in male and female mice begin to diverge after this juncture^7,8^. As murine pyelonephritis affects regions of the kidney unequally, we utilized the snRNAseq data to distinguish healthy cells from those affected by the experimental UTI. Analysis of sex differences in transcriptional programs in the collecting duct and proximal tubule revealed that females have a more focused response to infection, with marked upregulation of differentially expressed genes (DEGs) involved in pro-inflammatory transcription factor regulons, and KEGG pathway enrichment limited to medullary principal cells or injured proximal tubules. Males, on the other hand, had a more diffused response to UTI, with upregulation of DEGs involved in pro-inflammatory pathways across many cell types, reducing delineation between healthy and affected cells. These data indicate that host sex drives a divergent response to UTI across the nephron and help to illuminate how females better control infection in this model while males are inclined toward chronic infection and renal scarring.

## RESULTS

### Whole-kidney single-nucleus RNA sequencing during active bacterial infection.

**Figure 1:**
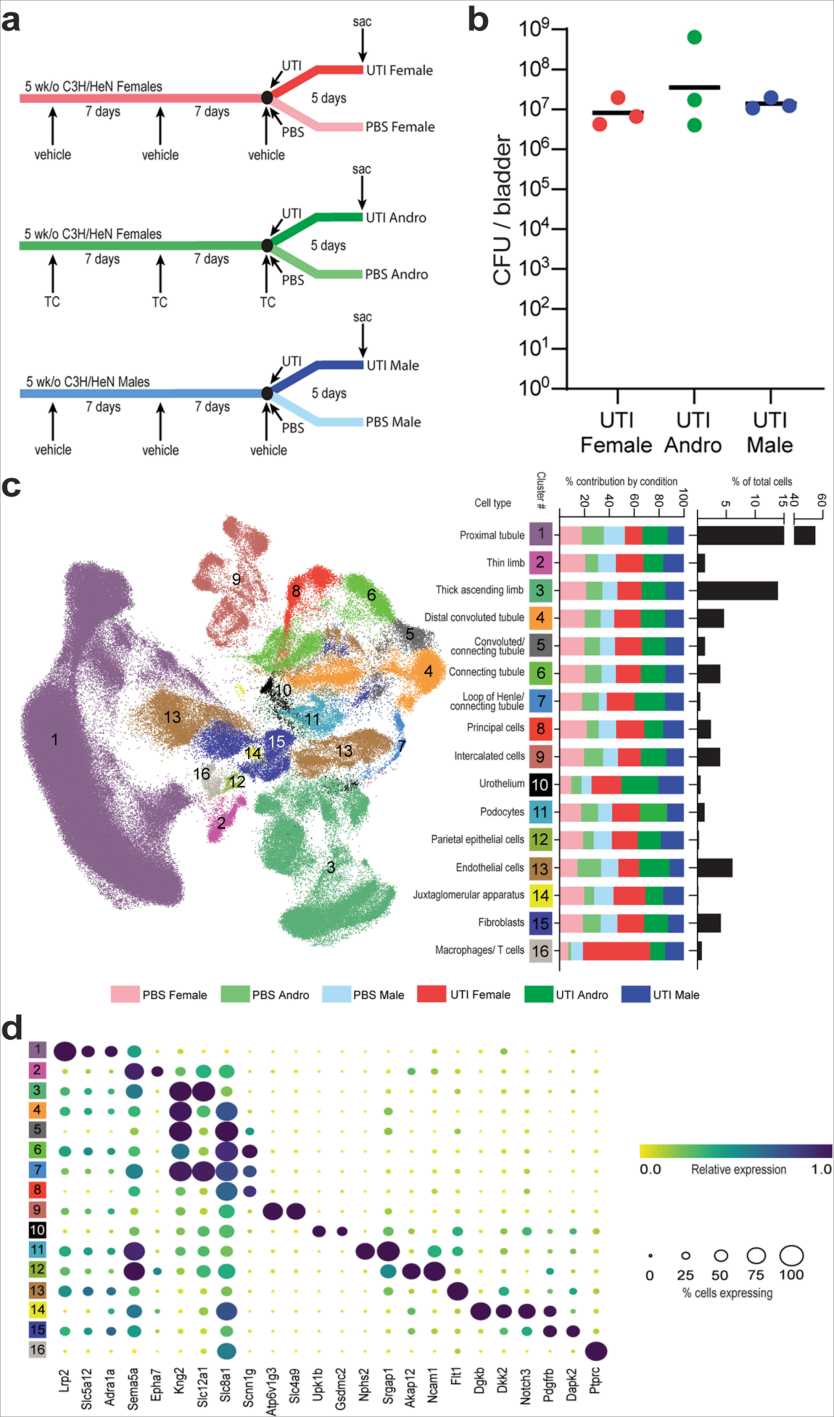
Experimental design and cell clustering. **a,** Schematic of experimental design and conditions in this study. Inoculations are indicated with arrows, including TC (testosterone cypionate); vehicle (cottonseed oil); UTI (UPEC strain UTI89 in PBS); or PBS. **b,** Bladder titers of the UTI mice selected for kidney single-nucleus RNA sequencing. **c,** Cluster designations and UMAP diagram for the 16 kidney cell types observed. Bar plots to the right of each cluster number indicate the proportional contribution to each cluster by each experimental condition (with red, green, and blue coloring as in (**a**)); the rightmost column indicates the percent contribution of each cluster to the total number of cells in the dataset. **d,** Marker genes identifying the 16 kidney cell types. Circle color indicates relative expression for each gene, while circle size indicates the percent of cells expressing this gene.

snRNAseq was performed on 18 C3H/HeN mice, three from each of six conditions. In the androgen-exposed (Andro) group, female C3H/HeN mice were injected with testosterone cypionate (TC) before the onset of UTI, as described previously^8^, while age-matched female and male C3H/HeN mice were injected with cottonseed oil. Mice at 7 weeks of age were inoculated intravesically with UPEC (UTI) or PBS (control) and sacrificed 5 days later (Fig. 1a). Three mice from each UTI group were selected for their high and comparable bladder bacterial loads (Fig. 1b) and visible renal abscesses at the time of sacrifice; three mice from each control (PBS) group were confirmed to have sterile bladders at the time of sacrifice. Nuclear isolation was performed as described previously^14,15^. Quality control and filtering of sequencing data included the use of CellBender^16^ to remove ambient RNA contamination, Scrublet^17^ to remove doublets, and Harmony data integration^18^ to correct for batch effects. We obtained sequencing data for a total of 248,483 high-quality single nuclei, representing 41,413 ± 6,005 cells per condition, with counts ranging from 4,895 – 18,106 cells per mouse. Cells were distributed across 16 expected kidney cell types, defined in part by marker gene expression, as in earlier reports^10,11^ (Fig. 1c, d), and were distributed relatively evenly across conditions, except that the UTI conditions contributed disproportionally to the Urothelium and Macrophage/T cell clusters (Fig. 1c). Recovered immune cells comprised only macrophages and T cells, lacking neutrophils as expected with single-nucleus isolation (as previously reported^10,11^). Of note, one female mouse with UTI contributed disproportionately to this cluster.

### Sex heavily influences transcription at the whole-kidney level.

**Figure 2:**
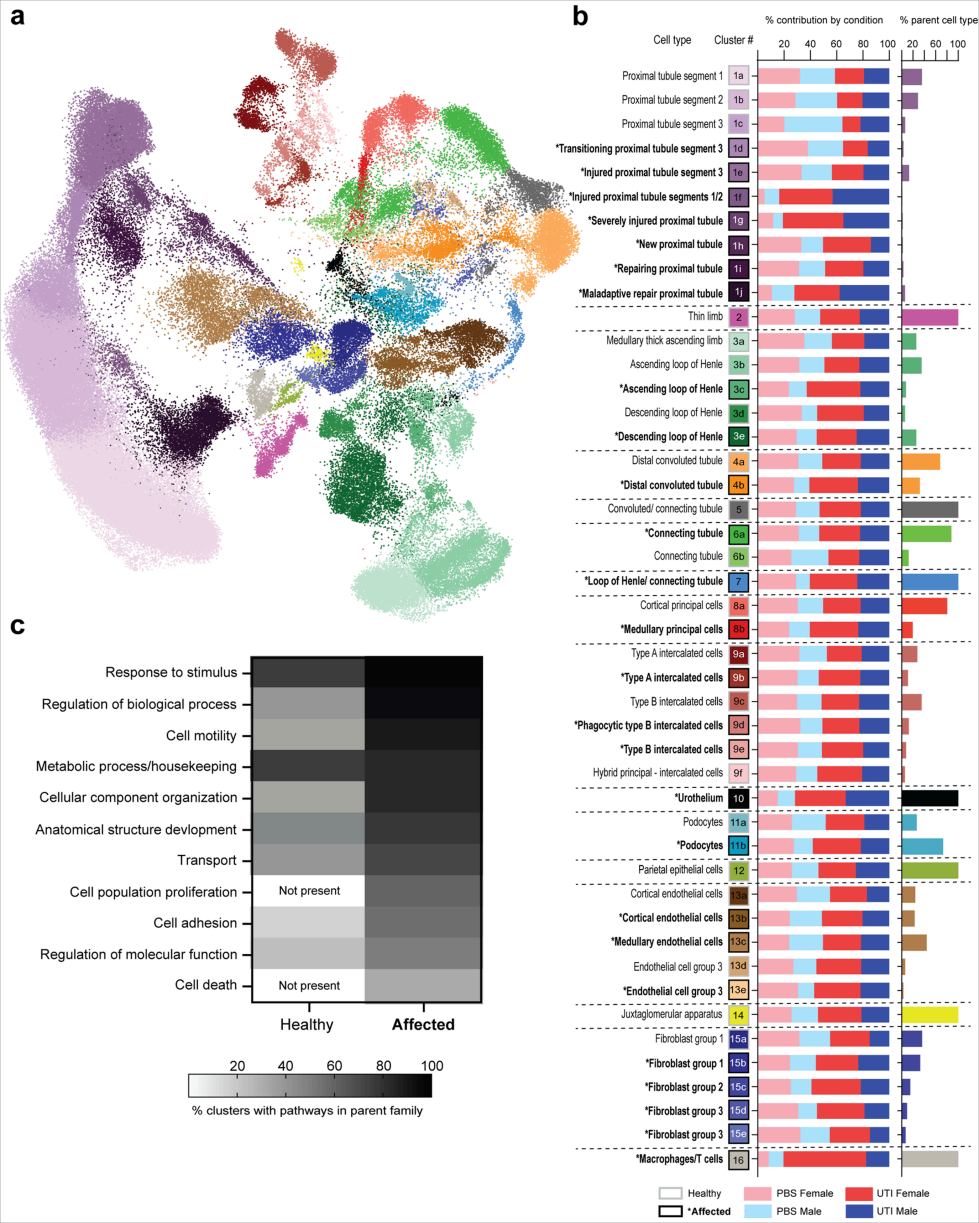
Influence of sex and exogenous testosterone on the kidney. **a,** Expression of sex-linked genes in female (red), Andro (green), and male mice (blue). Circle color indicates relative expression for each gene, while circle size indicates the percent of cells expressing this gene. **b,** Relative activity of the most differentially active TF regulons in each condition, based on comparisons of outputs from pySCENIC. The superset of the five most upregulated regulons during UTI in each condition are plotted. **c,** The number of significantly upregulated DEGs (*z*-score > 2.0) in each condition compared to all other conditions. **d,** The five most enriched KEGG pathways, based on the 200 most upregulated DEGs in female, Andro, and male mice are categorized by parent family.

To first verify that we could observe expected trends related to sex, we analyzed bulk transcriptional patterns of sex-linked and androgen-responsive genes. As expected, males exclusively expressed the Y-linked transcript *Uty* and exhibited the highest expression of androgen receptor (*Ar*) and the androgen-dependent transcripts *Fkbp5* and *Kap* (Fig. 2a). Furthermore, transcriptional analysis in androgen-exposed female (Andro) mice revealed high expression of X-inactivation genes *Xist* and *Tsix*, similar to that in females, while expression of androgen-responsive genes *Fkbp5* and *Kap* was similar to that in males. Andro mice exhibited the lowest expression of *Ar*, reflecting receptor downregulation in response to the exogenous testosterone treatment^19^ (Fig. 2a).

To investigate overall trends in expression related to sex, we identified the most differentially active transcription factor (TF) regulons in each condition using pySCENIC^20^, which performs gene regulatory network reconstruction and assesses the activity of regulons in specific cell types. This analysis showed that UTI activated the *Klf6* regulon in both male and female mice, which is known to regulate epithelial-mesenchymal transition and podocyte survival in the kidney^21^ (Fig. 2b and **Extended Dataset 1**). TFs responsible for cellular maintenance, such as *Nr1i2, Sal1*, and *Pura*, were highly active in PBS-exposed female mice, while several pro-inflammatory regulons, such as those of *Stat1, Runx1*, and *Jun*, were highly activated during UTI (compared to PBS conditions). Interestingly, these same inflammatory regulons were already somewhat active in PBS-inoculated male mice, indicating that male sex may predispose the kidney toward inflammatory responses to even non-infectious perturbation. However, during UTI, activity of pro-inflammatory TFs such as *Stat1* and *Runx1* was upregulated less strongly in males than in females, in agreement with other reports that female mice exhibit a more robust acute inflammatory response to UTI^6,22^.

Aligned with this observation, UTI females exhibited the most significantly upregulated differentially expressed genes (DEGs; *z*-score > 2.0) compared to all other conditions (Fig. 2c). PBS males had more upregulated DEGs than UTI males, reflecting their transcriptional response to PBS exposure. Further, the five most enriched KEGG pathways in the 200 most upregulated DEGs in female mice (both PBS and UTI conditions) compared to both male and Andro mice reflected metabolism, organismal systems, and cellular processes, while male mice were enriched in disease pathways. This difference highlights the overriding effect exerted by sex on transcriptional regulation in the kidney, regardless of UTI status (Fig. 2d and **Extended Dataset 2**).

Meanwhile, KEGG pathway analysis in Andro mice indicated that the experimental androgen exposure exerted outsized influence on gene expression in these mice. The most active regulons in all Andro mice, including the male-biased TFs *Bcl6, Nr1h4*, and *Hnf1b*^23,24^, were similar in both PBS or UTI exposure and were distinct from those most enriched in both male and female UTI (Fig. 2b and **Extended Dataset 1**). Though some of these regulons were also expressed in male mice, it was to a much lesser extent. Moreover, Andro mice had significant enrichment of DEGs involved in KEGG pathways related to xenobiotics biodegradation and metabolism, and hormone and drug processing (Fig. 2d and **Extended Dataset 2**). This domination of transcriptional responses in Andro mice by hormone-processing pathways permeated through our downstream analyses, precluding reliable assignment of the effects of UTI in Andro mice. As a result, we included the Andro groups in all cell-clustering determinations but elected to exclude the Andro groups from subsequent analyses, focusing only on the female and male groups.

### Identification of cells affected by UTI.

**Figure 3:**
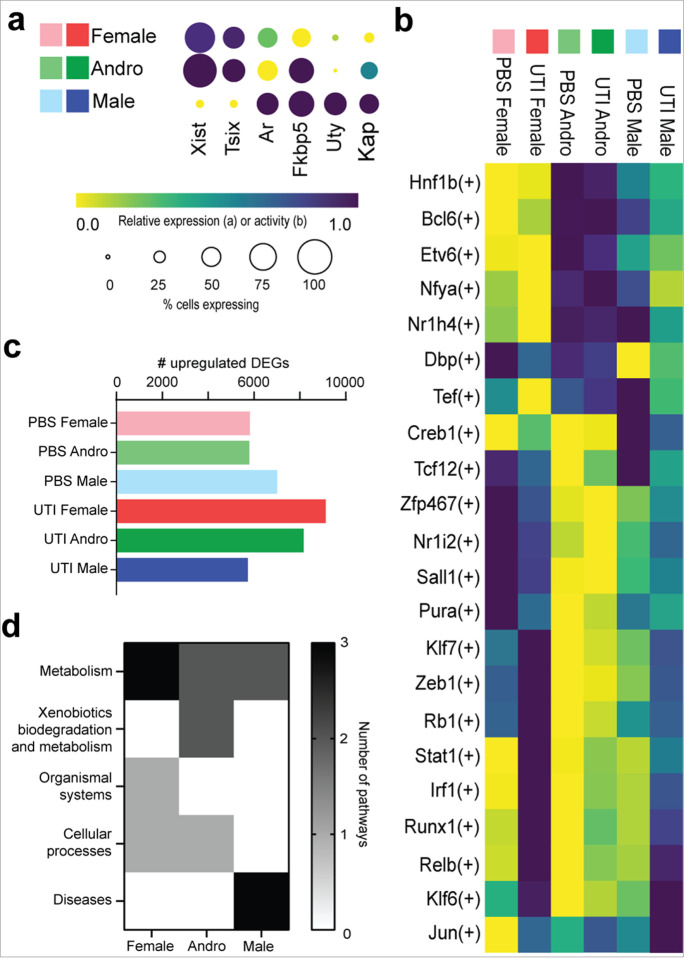
Distinguishment of healthy and affected clusters. **a,** UMAP of kidney cell types now further subdivided by cell type specificity as well as “healthy” or “affected” status. **b,** Cluster identification detailing the 46 clusters in the UMAP. In the left column, healthy cluster numbers have a gray bounding box, while affected clusters are indicated with an asterisk, bold labeling, and a black bounding box. In the middle column, the proportional contribution to each cluster by each experimental condition is indicated. Each cluster’s contribution to their parent cell type is shown in the rightmost column, colored by their parental cell type (from the 16-cluster scheme in Figure 1). **c,** The most significantly enriched GO terms, derived from the 200 most upregulated DEGs uniquely present in at least three healthy or injured clusters, are plotted as the percent of the total number of healthy or affected clusters with GO terms present in each of the represented parent families.

Because acute and chronic pyelonephritis in C3H/HeN mice is local and regional^7^ (i.e., not homogenous across the kidney as is seen in noninfectious injury models), it was important to leverage the snRNAseq dataset to distinguish **healthy** (apparently unaffected) cells from those **affected** by UTI or by the experimental perturbation of PBS inoculation. We increased the granularity of clustering in order to identify kidney cell subtypes^10–13,25–31^ and their affected status, yielding a set of 46 clusters (Figs. 3a, b and **Extended Data** Fig. 1). Proximal tubule (PT) subtypes (Fig. 3b, clusters 1a-j) were defined as described previously in experimental AKI^10^, with the addition of a new transitioning cluster (cluster 1d), which could not be defined as strictly healthy or affected in our dataset. Cells reflecting injured PT segments 1/2, severely injured PT, and PT undergoing maladaptive repair^10^ (clusters 1f, g, j) were observed predominantly in mice with UTI and were evenly distributed between females and males (Fig. 3b).

For the remaining cell types, excluding the Macrophages/T cells (cluster 16, which were classified as inherently affected), we identified healthy and affected subclusters using the Gene Ontology (GO) terms significantly enriched among the 200 most upregulated DEGs in each cluster compared to all others. The DEGs of affected clusters exhibited enrichment of GO terms such as response to stimulus, regulation of biological process, cell motility and transport (Fig. 3c and **Extended Dataset 3**). Of note, differences in GO term enrichment between healthy and affected cells were analogous to those reported between cells undergoing successful and maladaptive repair during noninfectious AKI^10^. Affected clusters also had many more enriched GO terms than healthy clusters (99 vs. 28, respectively; **Extended Dataset 3**). PBS-inoculated mice had cells present in every subcluster, and many affected clusters were similarly contributed to by UTI and PBS mice (Fig. 3b). This observation may reflect a baseline injury-like response due to the vesicoureteral reflux inherent to C3H/HeN mice, or a response to the inoculation event itself.

### Sex differences in the collecting duct response to UTI.

As ascending UPEC are known to interact directly with the collecting duct (CD) 5 dpi, forming kidney bacterial communities within the luminal space^7,32^, we examined in detail the effects of sex and UTI on CD cell populations. The eight CD subclusters (Fig. 3b) included principal cells (PCs; clusters 8a, b), intercalated cells (ICs; comprising IC-A [clusters 9a, b] and IC-B cells [clusters 9c-e]), and hybrid PC-IC subtypes (cluster 9f). Among PCs, only medullary (m) but not cortical (c) PCs could be classified as affected (Fig. 3b). While the phagocytic capacity of IC-A cells has been reported previously^26^, we identified a population of IC-B cells expressing phagocytic markers including *Dab2, Lrp2*, and *Cubn* (cluster 9d), indicating that these cells may also be able to phagocytose UPEC (**Extended Data** Fig. 2a).

**Figure 4:**
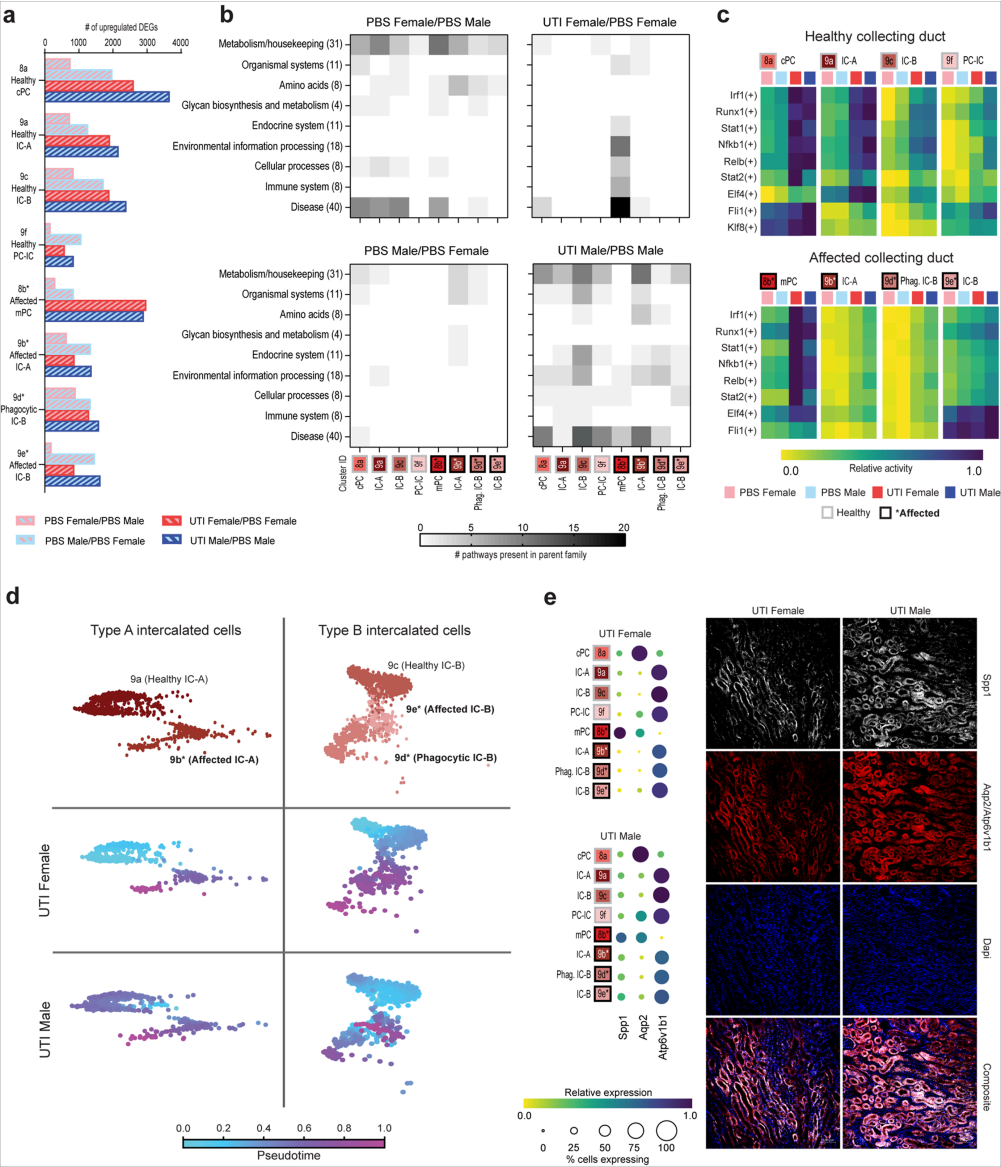
Sex differences in the collecting duct during UTI. **a,** The number of significantly upregulated DEGs (*z*-score > 2.0) in each CD subtype, shown for each pairwise comparison as indicated. **b,** Heatmaps depicting the number of KEGG pathways within each parent family that were significantly enriched among the 200 most upregulated DEGs when comparing the first versus the second condition indicated for each panel. **c,** Activity for the most differentially active TF regulons in each CD cluster during UTI. The upper and lower heatmaps show healthy and affected cell types, respectively. For each row, the superset of the five most differentially active regulons during UTI in each cell type are plotted. **d,** Pseudotime analysis of type A (left) and type B (right) intercalated cells during UTI. The top row shows the data colored by cell type; the bottom two rows show pseudotime calculations rooted at each healthy cluster, with fuchsia representing cells that are most different from the root. **e,** Relative expression of *Spp1, Aqp2*, and *Atp6v1b1* in the CD of UTI female and male mice (left), with corresponding IF microscopy images (right). cPC, cortical principal cells; mPC, medullary principal cells; IC-A, type A intercalated cells; IC-B, type B intercalated cells; PC-IC, hybrid principal-intercalated cells; Phag., phagocytic.

We first compared CD cell types in PBS-exposed female and male mice in order to examine sex differences at baseline. PBS male mice had more significantly upregulated DEGs compared to PBS females in every CD cluster (Fig. 4a). While the 200 most upregulated DEGs in PBS females (compared to PBS males) reflected KEGG pathways related to metabolism, amino acids, and disease, the DEGs in PBS males had few enriched KEGG pathways (Fig. 4b and **Extended Dataset 4**). Meanwhile, by pySCENIC analysis, healthy IC-A and IC-B clusters in PBS males had increased activity in inflammatory TF regulons (e.g., *Runx1, Irf1, Stat1, Nfkb1*) compared to PBS females (Fig. 4c and **Extended Dataset 5**), indicating that males may be predisposed to injury responses at baseline (e.g., related to VUR) or that they had an exaggerated response to sterile inoculation.

We then compared CD gene expression in males or females with UTI, to mice of the same sex without UTI. In both male and female mice, DEG upregulation related to UTI was most notable in PCs. UTI males had more significantly upregulated DEGs than UTI females throughout the CD, other than in mPCs (cluster 8b; Fig. 4a). Further, among KEGG pathways enriched in the 200 most upregulated DEGs during UTI in females, most were present in mPCs (Fig. 4b, upper right panel). These pathways included several involved in infection response, including environmental information processing, cellular processes, immune system and disease (Fig. 4b and **Extended Dataset 4**). Interestingly, pathways in these families were downregulated during female UTI (compared to PBS) in injured IC-As (cluster 9b; **Extended Data** Fig. 2b), further indicating that mPCs are a primary driver of the infection response in the female CD. In males, UTI provoked a broader response across all CD cell types (Fig. 4b, lower right panel); healthy and affected clusters exhibited similar patterns of KEGG pathway enrichment, whether in pathways involved in cellular homeostasis or in those reflecting cell stress or infection response.

Further, the most active TF regulons in the CD during UTI, based on pySCENIC analysis, also indicated a more dramatic response in affected mPCs (cluster 8b) in females. UTI males had comparatively less activity than UTI females in regulons corresponding to proinflammatory TFs such as *Irf1, Stat1*, and *Nfkb1*, except in affected IC-A and IC-Bs (clusters 9b and 9e) (Fig. 4c and **Extended Dataset 5**). Significantly downregulated TF regulons are shown in **Extended Data** Fig. 2c.

We next analyzed sex differences in the IC clusters of infected mice by pseudotime analysis, which enables representation of progression through a biological process (here infection-related injury) from transcriptomic data^33^. Analyses were rooted in the healthy IC-A and IC-B clusters and performed separately for female and male UTI conditions. Among IC-As (Fig. 4d, left), affected cells were more clearly differentiated from healthy cells along this injury trajectory in females, compared with males. A similar sex difference was evident in IC-Bs; while the trajectory was clear in females, males evidenced less demarcation between healthy and affected cells, or between these clusters and phagocytic IC-Bs (Fig. 4d, right). Taken together, the above set of analyses indicate that CD cell populations respond distinctly to UTI depending on sex, even in the context of equivalent bacterial loads.

Of additional note, genes involved in the KEGG pathway for extracellular matrix (ECM)-receptor interaction (contained within the environmental information processing family) were significantly enriched during male UTI in all but mPC, healthy IC-A and PC-IC clusters (clusters 8b, 9a, and 9f) but were expressed only in mPCs during female UTI (**Extended Dataset 4**). One of the genes involved in this pathway, *Spp1*, has been implicated in renal fibrosis by promoting Smad2/3 phosphorylation and promoting myofibroblast activation^34^, and overexpression of *Spp1* increased renal cell apoptosis during AKI^35^. Indeed, UTI male mice exhibited increased *Spp1* expression throughout the CD compared to UTI females, and immunofluorescence imaging showed more intense Spp1 staining throughout the CD in UTI males compared to UTI females (Fig. 4e). These data suggest that increased *Spp1* expression in the infected male kidney may play a role in the increased prevalence of renal scarring seen in these mice^7,9^.

### Sex differences in the proximal tubule response to UTI.

Since proximal tubule (PT) injury has been studied extensively in noninfectious AKI^10–13^, we investigated how sex influences transcriptional activity in PT in the absence and presence of infection. As noted earlier, cells from both female and male mice with UTI were overrepresented in injured PT segments 1/2 (cluster 1f), severely injured PT (cluster 1g) and maladaptive repair PT (cluster 1j) (Fig. 3b), indicating that UTI can drive PT injury responses, even if UPEC have not definitively reached this segment of the tubule.

**Figure 5:**
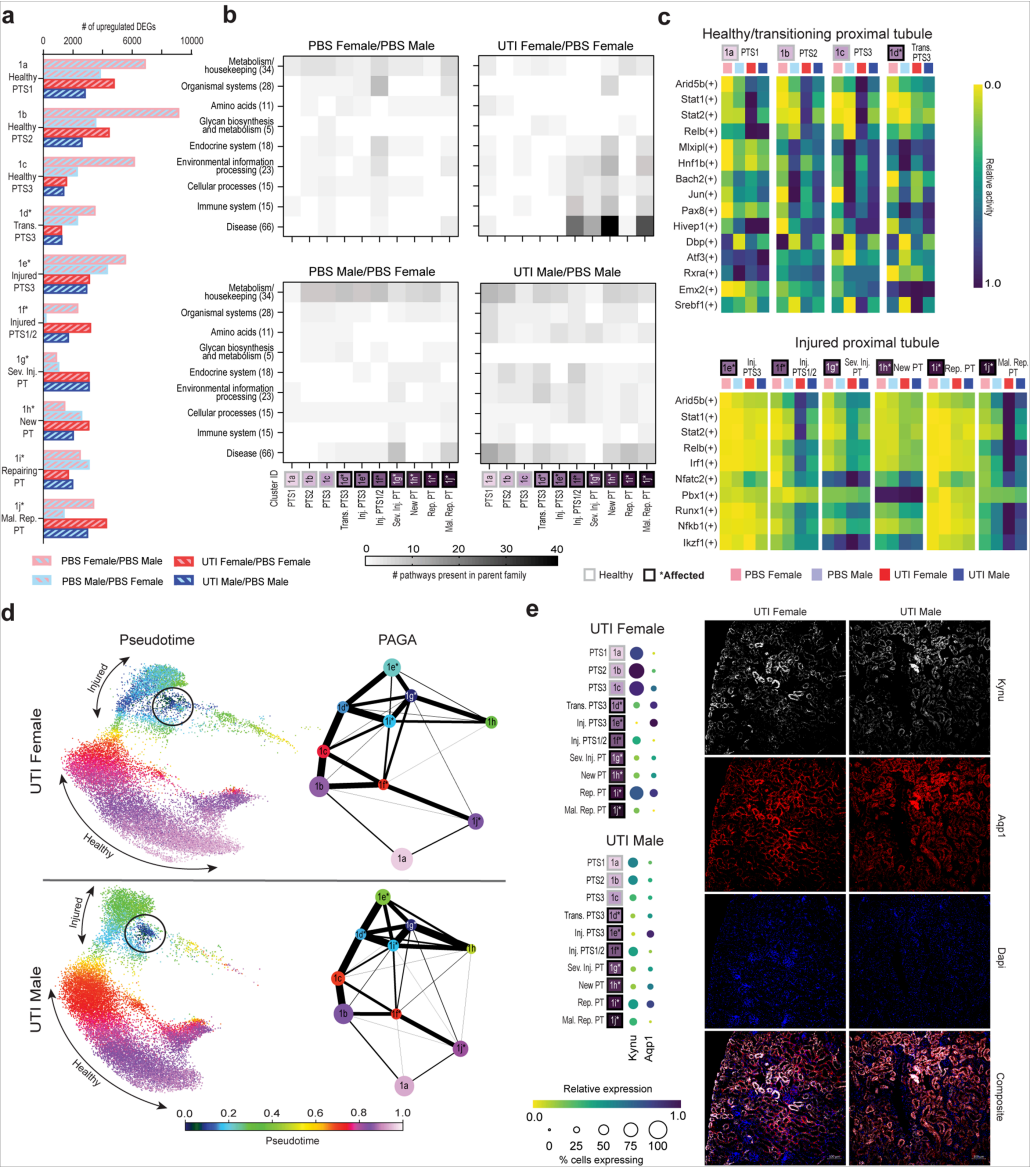
Sex differences in the proximal tubule during UTI. **a,** The number of significantly upregulated DEGs (*z*-score > 2.0) in each PT subtype, shown for each pairwise comparison as indicated. **b,** Heatmaps depicting the number of KEGG pathways within each parent family that were significantly enriched in the 200 most upregulated DEGs, when comparing the first versus the second condition indicated for each panel. **c,** Activity for the most differentially active TF regulons in each PT cluster during UTI, normalized by row. The upper and lower heatmaps show healthy/transitioning and injured PT cell types, respectively. For each row, the superset of the five most differentially active regulons during UTI in each cell type are plotted. **d,** Pseudotime (left) and PAGA (right) analysis of the proximal tubule during UTI. Pseudotime trajectories were rooted at the severely injured cluster (black circles), with light purple representing cells that are most different from the root. For PAGA analysis, the line weights represent connectivity between cell types. **e,** Relative expression of *Kynu* and *Aqp1* in the PT of UTI female and male mice (left), with corresponding IF microscopy images (right). PT, proximal tubule; S, segment; Trans., transitioning; Inj., injured; Sev. Inj., severely injured; Rep., repairing; Mal. Rep., maladaptive repair.

When sex differences between PBS conditions were examined, females had more upregulated DEGs than males in all PT clusters other than severely injured, new and repairing PT segments (Fig. 5a). Further, PBS females (compared to PBS males) exhibited the most upregulated DEGs among all conditions in all healthy PT segments, along with transitioning and injured PTS3 cells (clusters 1a-1e). However, these DEGs in PBS females comprised low involvement in any KEGG pathway family; the most involvement was observed in KEGG pathways related to metabolism and housekeeping, albeit to a lesser degree than in PBS males (Fig. 5b, left panels, and **Extended Dataset 6**). In the pySCENIC analysis, PBS males exhibited increased TF regulon activity in most cell clusters, compared to PBS females (Fig. 5c and **Extended Dataset 7**). This was particularly evident in healthy clusters, for example in the *Jun* regulon, well-studied in the immediate-early response process^36^. *Jun* activation in PBS males (reflecting activity at baseline or in response to sterile inoculation) was higher than that of UTI females in most healthy and transitioning PT clusters, consistent with a predisposition to inflammation and injury upon perturbation in the male kidney.

We next examined sex-dependent PT responses to UTI by comparing gene expression in PT cell types in males and females with UTI, to mice of the same sex without UTI. Unlike in the CD, UTI females exhibited more upregulated DEGs than males in many PT subtypes (Fig. 5a). However, as was seen in the CD, the PT in females evidenced a more cell-type-restricted response to UTI. Specifically, many injured or regenerating cell clusters (clusters 1f-h and 1j) showed significant upregulated DEGs involved in KEGG pathways relating to environmental information processing, cellular processes, immune system and disease, while pathways from these same families were comparatively downregulated during UTI (compared with PBS females) in other PT clusters (1a-e and 1i) (Fig. 5b, upper right panel, and **Extended Dataset 6**). Further, considering the most active TF regulons in UTI based on pySCENIC analysis (regardless of sex), UTI females showed the most activity in injured PT clusters, with the most significant regulon activity in injured segments 1/2, severely injured PT, and maladaptive repair PT (clusters 1f, g, j; Fig. 5c and **Extended Dataset 7**). Significantly downregulated TF regulons are shown in **Extended Data** Fig. 3a.

In males with UTI (compared to PBS males), the most upregulated DEGs represented more modest enrichment of KEGG pathways across the array of PT cell types, indicating (as seen in the CD) a broader, less cell-type-specific response to UTI than in females (Fig. 5b and **Extended Dataset 6**). DEGs involved in KEGG pathways reflecting disease were more expressed across virtually all PT clusters during male UTI, in contrast to the cell-type restriction in females. Interestingly, DEGs involved in disease-related KEGG pathways were significantly upregulated in healthy PT and injured PTS3 segments of PBS females compared to UTI females (**Extended Data** Fig. 3b). This effect was not seen in the same comparison in males, indicating that healthy PT cells in females may reduce this type of gene expression during UTI.

As we did in the CD, we performed separate pseudotime analyses of male and female PT clusters during UTI, rooting these analyses at the severely injured PT (cluster 1g; Fig. 5d, circled) to examine the relationships between injured cells and healthy or repairing clusters. Healthy PT cell clusters in male UTI (Fig. 5c, red/purple) demonstrated a more constrained trajectory and darker coloration than those in UTI females, indicating that healthy PT cells more closely resemble severely injured cells in UTI males. While the pseudotime trajectory showed injured PT clusters (Fig. 5d, blue/green) in UTI males as less related to severely injured cells than in UTI females, PAGA plots (a graphical abstraction that reconciles cell clustering with the trajectory inference^37^) showed closer relationships (denoted by thicker connecting lines) among injured clusters in infected males, as well as more connections between healthy and injured clusters (Fig. 5d, right). This abundance of relatedness between healthy and injured PT clusters may indicate that male sex primes the PT for an injurious response to UTI.

Finally, we examined the expression of kynureninase (*Kynu*) in the PT during UTI. The kynurenine pathway is known to represent an early kidney injury marker and is induced by cytokines such as IFNγ and TNFα^38^, which are produced at higher levels in UTI females at early time points^6,22^. Interestingly, while activation of the kynurenine pathway reduces neutrophil migration and promotes UPEC survival during UTI^39^, inhibition of this pathway promotes renal fibrosis in AKI by enhancing epithelial-to-mesenchymal transition^40^. Our results demonstrated increased *Kynu* expression in UTI females compared to UTI males, especially in healthy and repairing PT clusters (Fig. 5e). UTI males, in contrast, failed to increase *Kynu* expression in injured PT (compared with healthy PT clusters). Immunofluorescence imaging of the PT reflected these results, with UTI female mice demonstrating regions of both high and low Kynu positivity, while UTI males showed more diffuse staining and increased colocalization with Aqp1 throughout the PT (Fig. 5e). Further, examination of the kynurenine aminotransferases (KATs) involved in synthesizing kynurenic acid, which acts as an endogenous inhibitor of the kynurenine pathway^38^, revealed that UTI females had higher expression of KAT II (*Aadat*) and III (*Kyat3*) in injured PT clusters, while UTI male mice had expression of KAT I (*Kyat1*) and II throughout the PT, with lower expression of KAT II and III compared to females (**Extended Data** Fig. 3c).

In UTI males, expression of these KAT genes mirrored that of *Kynu*, indicating that these cells may be actively inhibiting this pathway. These findings are consistent with a model in which early high-level expression of *Kynu* in PT in females (as opposed to the more moderate, stable expression in males) acts to limit UTI-induced injury.

### Sex differences in cell-cell communication during UTI.

**Figure 6:**
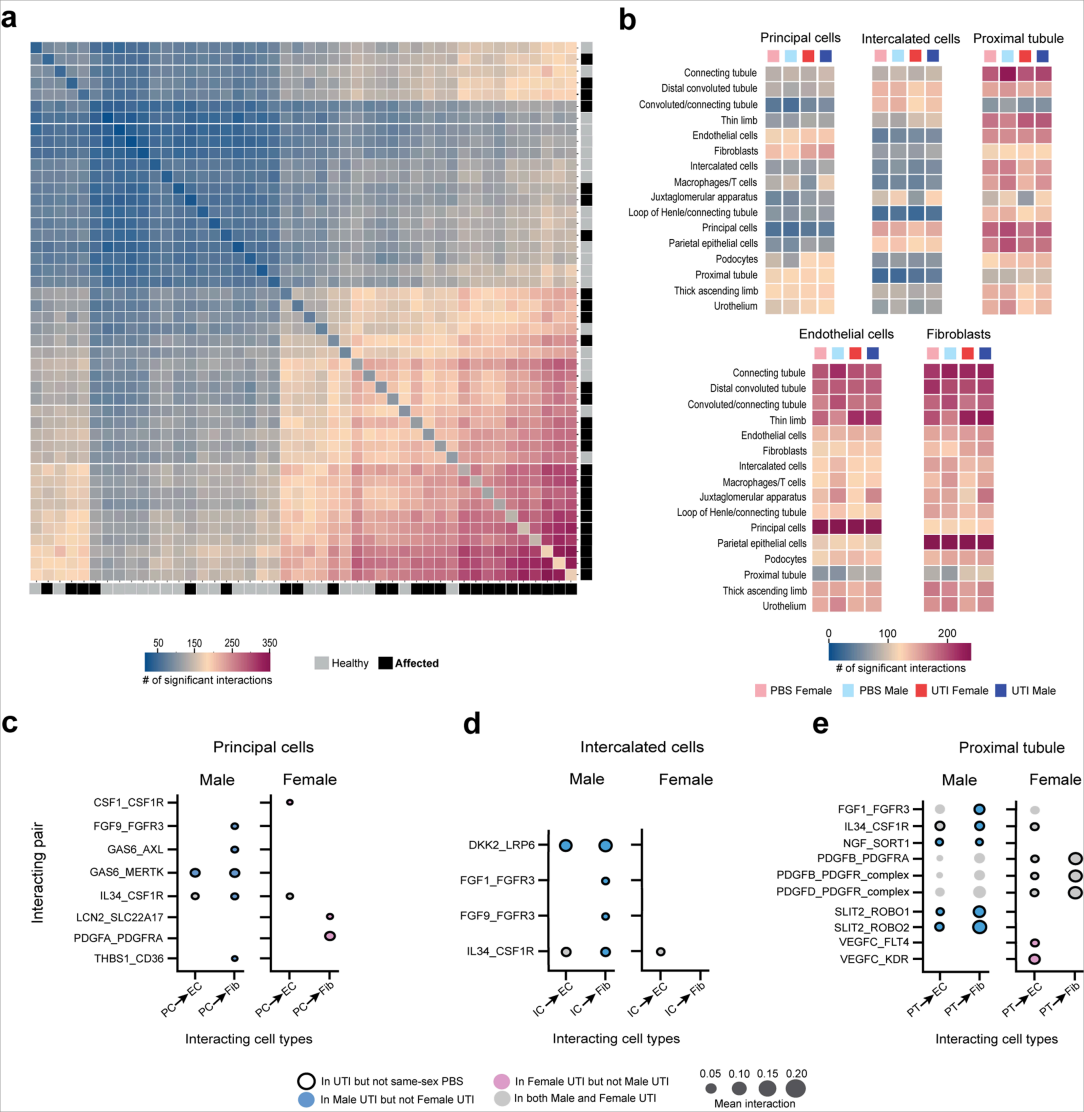
Sex differences in cell-cell communication during UTI. **a,** Heatmap of the number of significant cell-cell interactions based on CellPhoneDB analysis, demonstrating that healthy clusters (gray boxes along right and lower borders) exhibit fewer interactions than injured clusters (black boxes along right and lower borders). **b,** Heatmaps of the number of significant cell-cell interactions with ligands produced by principal cells, intercalated cells, proximal tubule cells, endothelial cells, and fibroblasts to receptors expressed on other kidney cell types. **c-e**, Cell-cell interactions of ligands secreted by principal cells (**c**), intercalated cells (**d**), and proximal tubule cells (**e**) to receptors on endothelial cells (EC) or fibroblasts (Fib) that were unique to males or females during UTI.

At 5 dpi, UPEC are known to interact directly with CD cells^7,32^, but we found injury signals throughout the nephron (including in PT) during UTI. We therefore further interrogated this finding by analyzing cell-cell communication among all 46 clusters using CellPhoneDB^41^, which allows inferences regarding cell-cell communication by examining the combined expression of ligand-receptor complexes. Overall, affected cell clusters were more frequently involved in measurable communication than healthy clusters (Fig. 6a). When examining the number of cell-cell interactions in only the 16 basic kidney cell types (not subdivided into healthy or affected clusters), we found that these interactions were more influenced by sex than by UTI status. In other words, the PBS and UTI-exposed mice of each sex displayed the most comparable numbers of cell-cell interactions. Within the CD, ICs showed relatively few significant communications, interacting mostly with nearby cells such as PCs and DCTs (Fig. 6b). PCs exhibited more frequent and broader interactions, including with endothelial cells (ECs), fibroblasts (Fibs), and PT cell types (Fig. 6b), suggesting that PCs may be chiefly responsible for communication from the CD to the rest of the kidney.

We then examined sex differences in specific interactions between ligands secreted by ICs, PCs, or PT cells to surface-expressed receptors on ECs and Fibs, as activation of these latter cell types drives the renal response to UTI and promotes scarring^42,43^. The interaction set was refined to include only interactions featuring a secreted ligand and with at least one instance of being uniquely present in either the male or female UTI condition (Fig. 6c–e and **Extended Dataset 8**). Males exhibited more unique interactions during UTI, and this refinement resulted in no interactions by ICs that were specific to UTI females (Fig. 6d). During UTI, males showed unique interactions relating to pro-fibrotic wound healing responses, including mesenchymal stem cell activation through TGF 1 superfamily signaling^44–46^ (GAS6 and THBS1 by PCs; Fig. 6c) and Wnt signaling^47^ (DKK2 by ICs; Fig. 6d), as well as interactions relating to activation (FGF)^48^ and pro-inflammatory signaling (IL34)^49^ by both ICs and PT cells (Fig. 6d, e). Interestingly, PT cells in female mice participated in PDGFB/D_PDGFR interactions only during UTI (Fig. 6e, black outline), while such interactions were already present in PBS males (Fig. 6e, no black outline). These interactions on fibroblasts play a major role in renal fibrosis and scarring in AKI^50^; their occurrence even in uninfected males further underscores the increased propensity of males toward renal scarring during experimental pyelonephritis^9^. Potentially moderating this pro-fibrotic tendency in males, UTI male PTs had unique interactions with ECs and Fibs through SLIT2 and ROBO1/2 (Fig. 6e). These interactions have been shown to modulate actin rearrangement in a variety of cell types and are proposed to exert anti-fibrotic action in AKI^51^.

The unique cell-cell interactions evident in UTI females, rather than reflecting scarring propensity as in males, instead illuminated pathways of repair and defense. Though UTI female PCs uniquely interacted with Fibs through PDGFA (Fig. 6c), PDGFA is thought to be much less involved in fibrosis compared to PDGFB and PDGFD^52^. Further, UTI female PCs highly expressed CSF1, which can induce a cytokine cascade via ECs to promote activation of reparative macrophages^53,54^. UTI female PCs also secreted LCN2, which limits bacterial growth by sequestering iron during UPEC cystitis^55^ (Fig. 6c). Finally, sex differences in PT and EC communication may also affect cell survival. UTI males were found to participate in NGF_SORT1 signaling, which can promote apoptosis^56^, while UTI females more highly evidenced VEGFC_FLT4/KDR signaling, which promotes EC survival and proliferation^57^ (Fig. 6e). Taken together, these patterns of unique cell-cell communication in infected mice further support a model in which males are predisposed to aberrant wound healing and scarring, while females are more successful in antibacterial defense and repair.

## DISCUSSION

Single-cell (sc) and single-nucleus (sn) RNA sequencing has been used extensively to study the kidney response to many types of acute and chronic injuries and perturbations^58,59^. In this study, we used snRNAseq to investigate how sex influences the transcriptional response of the kidney during high-titer pyelonephritis, creating a dataset consisting of nuclei isolated from the kidneys of female, male, and androgen-exposed female C3H/HeN mice 5 days following intravesical inoculation with UPEC strain UTI89 or with PBS (control). At this timepoint, UPEC are thus far known only to directly interact with the collecting duct, particularly with Aqp2^+^ medullary principal cells^32^. Further, pyelonephritis in these mice does not affect the whole kidney homogeneously, instead resulting in regional foci of infection and abscess formation surrounded by areas of healthy tissue^7^. Specifically, at 5 dpi, UPEC kidney bacterial communities are just beginning to form^7,32^ and abscess formation is limited. These facts have curtailed our ability to use traditional whole-kidney analyses to understand early kidney responses to UTI and their sex specificity.

With our experimental design, we hoped to identify which gene expression differences between male and female mice were attributable to androgen action. Androgen-exposed (Andro) female mice had the same phenotypic response to UTI as males^6–9^, but the transcriptional profiles of both PBS and UTI-exposed Andro mice in this dataset were overwhelmingly dominated by genes and pathways involved in hormone responses. The Andro mice received testosterone cypionate for only two weeks before the initiation of UTI, and this interval may not have been sufficient to stabilize host responses to exogenous androgen exposure. Therefore, in this report we excluded the Andro mice from analyses beyond the initial cluster identification. The gene expression data from Andro mice are posted online along with the male and female mouse data described in this manuscript; additional post-publication analysis may or may not help to separate the effects of UTI from those of exogenous androgen exposure.

Using the snRNAseq dataset, we were able to successfully identify and distinguish cells affected by UTI (or by PBS inoculation) from cells that remained healthy. We were able to identify affected cells in almost all 16 kidney cell types, indicating that UTI exerts wide-reaching effects across the kidney and beyond those arising from direct UPEC-host cell interaction. Our cell-cell communication analysis revealed that affected clusters exhibited more interactions with other cells than did healthy ones, and when communication between specific cell types was examined, the cells of the CD (PCs and ICs) evidenced fewer cell-cell interactions than the PT overall, regardless of inoculation condition. Further, cluster assignment in the PT in our dataset closely mirrors that of PT injury during AKI^10^. These data indicate that although UPEC might not directly interact with the PT at 5 dpi, these cells are highly sensitive to UTI-related injury and actively participate in communication with multiple other cell types across the kidney.

We chose to focus our investigation on sex differences in the CD – where UPEC are known to directly interact with host cells 5 dpi^32^ – and the PT, where cellular injury and repair pathways have been very well described in noninfectious injury models^10,12^. In both of these nephron segments, males and females had a similar distribution of cells between healthy and affected clusters, but sex had a significant influence on transcriptional responses to UTI. Females exhibited a more targeted response to infection, with few cell types (e.g., mPCs and injured PT clusters) showing marked transcriptional regulation. In contrast, males had a more diffused response to UTI, with both healthy and affected clusters exhibiting upregulation of KEGG pathways and TFs related to disease and pro-inflammatory signaling. Overall, the male response to UTI also was less pronounced (of lower “amplitude”) than that in females, with UTI females having the highest relative activity of UTI-related TFs in both the CD and PT.

Previous data have shown that in general (through whole-kidney cytokine quantification, Western blotting or flow cytometry), males (or androgenized females) have higher tissue levels of pro-inflammatory cytokines, and increased pro-fibrotic signaling compared to females^6,8,9^. This phenomenon is evidenced in this snRNAseq dataset by significant expression of disease-related KEGG pathways in male mice regardless of inoculation condition. Males in our study also exhibited less defined pseudotime trajectories between clusters in CD and PT during UTI (i.e., more relatedness between healthy and affected clusters). Further, we examined the expression of *Spp1* in the CD and *Kynu* in the PT. Spp1 is known to induce TGFβ1-dependent myofibroblast activation through increased Smad2/3 phosphorylation^34^, and its expression in UTI females was largely restricted to mPCs, while males exhibited diffuse expression of *Spp1* throughout the CD, consistent with their phenotypic susceptibility to post-pyelonephritic scarring^7,9^. Meanwhile, in the PT, *Kynu* was most expressed in healthy clusters in females, while UTI males again exhibited broad but comparatively lower *Kynu* expression throughout the PT as well as expression of genes involved in *Kynu* pathway inhibition, aligning with reports linking kynurenine pathway inhibition to enhanced epithelial-to-mesenchymal transition in AKI^38,40^. Finally, in other studies examining earlier time points in murine UTI (1 dpi), the innate response in females was earlier and of higher amplitude, while the male response was comparatively dampened and delayed^6,22^. Thus, future single-cell analyses at earlier time points during male and female UTI might further elucidate why females control experimental UTI better than males.

Interestingly, cells from PBS mice were present in every affected cluster and contributed a similar number of cells to many of these clusters as did UTI-infected mice. The presence of affected cells in PBS mice may reflect responses to VUR inherent to the C3H/HeN strain or be related to the PBS inoculation itself. Regardless, transcriptional profiles in these affected clusters were very different between PBS and UTI. PBS males had increased activity of pro-inflammatory TFs in injured CD and PT clusters compared to PBS females, consistent with a propensity toward injury in male mice.

The divergence between male and female transcriptional responses to UTI evident in this dataset, even though these mice exhibited similar bacterial loads and early abscess formation at sacrifice 5 dpi, begins to illuminate the underlying basis for the markedly sex-discrepant outcomes of renal infection in this model^6,7^. The data do not designate a single pathway driving these outcomes; instead, it is clear that male sex exerts broad cellular effects on response to bacterial infection in the kidney, including through testosterone-dependent mechanisms^6,7^.

## METHODS

### Bacterial culture.

Uropathogenic *Escherichia coli* (UPEC) strain UTI89 was grown statically overnight in Luria-Bertani (LB; Becton Dickinson) broth at 37°C. Cultures were centrifuged at 7,500 × *g* at 4°C before being resuspended to a final density of ~ 4 × 10^8^ colony-forming units [CFU]/mL in sterile phosphate-buffered saline (PBS).

### Animals.

All animal studies were approved in advance by the Washington University Institutional Animal Care and Use Committee. For androgen exposure, 5-week-old female C3H/HeN mice (Envigo #040) were given weekly intramuscular injection of 150 mg/kg testosterone cypionate in cottonseed oil (TC; McKesson Medical) for 2 weeks before induction of urinary tract infection (UTI). All other mice received weekly injections of cottonseed oil. For induction of UTI, 7-week-old mice were inoculated transureuthrally with 1–2 × 10^7^ CFU of UTI89 in PBS or with sterile PBS as described previously^60–62^.

### Determination of bacterial loads.

At 5 dpi, mice were sacrificed via CO_2_ asphyxiation, and bladders and kidneys were aseptically removed. Bladders were homogenized in 4°C PBS, and homogenates were serially diluted and plated on LB agar. Individual kidneys were placed in 1.5-mL tubes and flash frozen in liquid nitrogen before storage at −80°C. As bladder and kidney bacterial loads are correlated^6,8^, chronic infection in UPEC-infected mice was defined as bladder titers above 10^6^ CFU/mL.

### Isolation of nuclei.

Kidney nuclear preparation was completed as described previously^14,15^. Briefly, flash-frozen kidneys were minced on ice into cold nuclei lysis buffer (Sigma #NUC101) supplemented with RNasin Plus (Fisher #PRN2615) and SUPERaseIN (ThermoFisher #AM2696), then homogenized using a Dounce homogenizer. 2 mL of lysis buffer was added to the homogenate before incubating for 5 min on ice, and the suspension was passed through a 40-μm strainer (Puriselect #43-50040-51) and centrifuged at 500 × *g* for 5 min at 4°C. The resulting pellet was washed with lysis buffer and incubated for 5 min at 4°C before being centrifuged again. The pellet was resuspended in nuclei suspension buffer (Dulbecco’s PBS [Sigma], RNasin Plus) and passed through a 5-μm strainer (Puriselect #43-50005-03) before nuclei were counted on a hemacytometer. Nuclei were diluted to a final concentration of 1200 nuclei/μL in 1% bovine serum albumin, 0.2 U/μL RNasin in DPBS and sequenced immediately.

### Sequencing.

A total of 18 samples were sequenced. Library prep, barcoding, and pooling were performed on the 10X Chromium platform. Sequencing was performed on an Illumina NovaSeq 6000 instrument. Among the 18 samples, two samples (PBS-inoculated males) were resequenced due to low nuclei counts or out-of-range ambient RNA content on initial sequencing.

### Analysis of single-nucleus RNA sequencing data.

Initial analysis of sequencing data was performed using 10X Genomics CellRanger software (version 6.0.1). CellBender^16^ (version 0.1.0) was used to remove ambient RNA signals, using stringent thresholds (CellBender expected number of cells 10,000; CellRanger Total_droplets_included 100%). Quality control, filtering, and downstream analyses were performed using Scanpy^63^ (version 1.9.3). In filtering steps, we required (i) ≥ 1200 genes/cell (chosen to obtain a realistic number of cells per dataset while minimizing noise; (ii) ≤ 5% mitochondrial genes; and (iii) that each gene be present in ≥ 3 cells. Analysis steps included normalization, log transform, identification of highly variable genes, and principal component analysis. Harmony^18^ (version 0.0.6) was used for integrating single-cell data from multiple experiments across both batch and condition. Doublets were removed with Scrublet^17^ (version 0.2.3). After computing a neighbor map, UMAP was used for dimensional reduction, followed by cell clustering using the leiden algorithm^64^. Genes differing between groups of cells were ranked using a t-test (scanpy method t-test_overestim-var). Cirrocumulus^65^ was used for visualization.

### Cell type clustering and annotations.

Cells were initially clustered into 98 clusters, using leiden clustering with the parameter “resolution = 5.0.” These 98 clusters were then manually grouped at three different levels of resolution: (i) 16 general cell types; (ii) 30 specific cell types; and (iii) 46 specific cell types, each split into healthy versus affected groups (**Extended Dataset 10**). In total, 6631 cells were removed from analysis. Cell type annotations were determined by manual inspection of marker genes and comparison to previous works^10–13,25–31^, and validated using integration mapping and automatic transfer of cell type annotations from a previous study of kidney cells^25^, using the label transfer approach in Seurat^66^ (version 4.3.0).

As the immune cell cluster (initially containing 4890 cells) initially appeared to also contain non-immune cells, it was manually clustered at higher resolution. Using the leiden algorithm in Scanpy with the parameter “resolution = 0.25,” we split these immune cells into five new clusters. Two of these new clusters were retained and combined into a single immune cell cluster, while the other three clusters (containing a total of 3482 cells) were discarded from analysis, as they did not represent immune cells. The final number of cells subjected to downstream analysis, across all conditions, was 248,483.

### KEGG pathway enrichment.

ShinyGO^67^ (version 0.80) was used to determine significantly enriched KEGG pathways (FDR < 0.05) from the 200 most upregulated DEGs by *z*-score for each of the comparisons described in Results. Parent families were determined by using the KEGG BRITE database^68^. All significantly enriched KEGG pathways for each of the cell types or conditions described in the results were analyzed together, with the figures representing the number of KEGG pathways from any cluster in a given parent family that are present in a specific cluster or condition.

### GO term enrichment.

GO enrichment analysis was performed using the Gene Ontology Database^69–71^ from the 200 most upregulated DEGs by *z*-score in a given healthy or affected cluster compared to all other clusters. Parent families were determined using AmiGO^72^. Differences in GO term enrichment in healthy and affected clusters were limited to significantly enriched GO terms (FDR < 0.05) terms that were present in at least three healthy or affected clusters, while also present in at least three of those clusters. GO terms identifying processes outside of the kidney were also removed.

### Additional downstream analyses.

Pseudotime analysis was performed separately for each experimental condition using Diffusion Pseudotime^33^ in Scanpy, and PAGA was used to generalize relationships between cell types^37^. Regulon analysis was performed using pySCENIC^20^ (version 0.12.1). For pySCENIC, we re-ran the Scanpy pipeline keeping all genes, including rare genes. Using pySCENIC outputs, we performed comparisons between conditions as we did for expression data, using a t-test. Analysis of cell-cell communication was performed separately for each experimental condition using CellPhoneDB^41^ (version 5.0.0). To use CellPhoneDB, mouse cells were mapped onto human cells using pre-computed homologs from MGI^73^ version 6.18.

### Immunofluorescence microscopy.

For immunofluorescence studies, mice were anesthetized with isoflurane before perfusion with cold, sterile PBS. Kidneys were decapsulated, bisected, and fixed in 4°C 4% paraformaldehyde in PBS before incubation in 30% sucrose overnight at 4°C. Fixed kidneys were then mounted in OCT (Tissue-tek) before cryosectioning into 5–8 μm sections and mounting on Superfrost microscope slides. Sections were rinsed with PBS to remove the OCT, permeabilized with 0.25% Triton X-100 (Sigma) in PBS for 10 min, then blocked for 1 h with 10% fetal bovine serum (Gibco) in PBS before staining with primary antibodies against aquaporin-2 (Aqp2-AlexaFluor 488 for PCs; Santa Cruz Biotechnology #sc-515770), V-type protein ATPase subunit B (Atp6v1b-AlexaFluor 488 for ICs; Novus Biologicals #NBP2–70237AF488), aquaporin-1 (Aqp1 for PT; Santa Cruz Biotechnology #sc-25287, secreted phosphoprotein 1 (Spp1-MFluor Violet 610; Novus Biologicals #NBP-20774MFV610), or kynureninase (Kynu; ThermoFisher #PA593121) in blocking buffer. Slides were washed with PBS; for Aqp1 and Kynu-stained slides, secondary staining with goat anti-mouse-AlexaFluor488 (Molecular probes #A-21121) and donkey anti-rabbit-AlexaFluor 594 (Jax Immuno #711-585-152) was performed, followed by an additional PBS wash. Slides were stained with 1:5000 4’,6-diamidino-2-phenylindole (DAPI) in PBS for 5 min, washed and mounted with Prolong Gold Antifade Reagent (ThermoFisher Scientific #P36930). Stained slides were imaged with a Zeiss LSM 880 Airyscan confocal microscope (Oberkochen, Germany).

## Supplementary Files

This is a list of supplementary files associated with this preprint. Click to download.

- ExtendedDataTH62525.pdf

## Data Availability

Gene expression data have been submitted to the Gene Expression Omnibus (GEO) under accession number GSE296327, and Illumina reads have been submitted to NCBI’s Short Read Archive (SRA) under NCBI bioproject PRJNA1182331.
